# iTRAQ-Based Proteomic Analysis Reveals Several Strategies to Cope with Drought Stress in Maize Seedlings

**DOI:** 10.3390/ijms20235956

**Published:** 2019-11-26

**Authors:** Zhilei Jiang, Fengxue Jin, Xiaohui Shan, Yidan Li

**Affiliations:** 1 Institute of Agricultural Biotechnology, Jilin Academy of Agricultural Sciences/Jilin Provincial Key Laboratory of Agricultural Biotechnology, Changchun 130033, China; jiang1891@aliyun.com (Z.J.); fxjin1973@126.com (F.J.); 2 College of Plant Science, Jilin University, Changchun 130062, China

**Keywords:** iTRAQ, proteomics, drought stress, differentially accumulated protein species (DAPS), *Zea mays* L.

## Abstract

Drought stress, especially during the seedling stage, seriously limits the growth of maize and reduces production in the northeast of China. To investigate the molecular mechanisms of drought response in maize seedlings, proteome changes were analyzed. Using an isotopic tagging relative quantitation (iTRAQ) based method, a total of 207 differentially accumulated protein species (DAPS) were identified under drought stress in maize seedlings. The DAPS were classified into ten essential groups and analyzed thoroughly, which involved in signaling, osmotic regulation, protein synthesis and turnover, reactive oxygen species (ROS) scavenging, membrane trafficking, transcription related, cell structure and cell cycle, fatty acid metabolism, carbohydrate and energy metabolism, as well as photosynthesis and photorespiration. The enhancements of ROS scavenging, osmotic regulation, protein turnover, membrane trafficking, and photosynthesis may play important roles in improving drought tolerance of maize seedlings. Besides, the inhibitions of some protein synthesis and slowdown of cell division could reduce the growth rate and avoid excessive water loss, which is possible to be the main reasons for enhancing drought avoidance of maize seedlings. The incongruence between protein and transcript levels was expectedly observed in the process of confirming iTRAQ data by quantitative real-time polymerase chain reaction (qRT-PCR) analysis, which further indicated that the multiplex post-transcriptional regulation and post-translational modification occurred in drought-stressed maize seedlings. Finally, a hypothetical strategy was proposed that maize seedlings coped with drought stress by improving drought tolerance (via. promoting osmotic adjustment and antioxidant capacity) and enhancing drought avoidance (via. reducing water loss). Our study provides valuable insight to mechanisms underlying drought response in maize seedlings.

## 1. Introduction

Drought is a major environmental factor affecting crop production. With global warming, the frequency of drought has also increased significantly. Maize (*Zea mays* L.), as an important crop, is often affected by drought or moisture deficit. Drought has seriously threatened maize production worldwide, especially under rain-fed conditions [1]. In China, more than 70% of maize growing areas are threatened by drought stress [2]. In the northeast of China, drought mostly occurs in spring and affects maize seedling growth and yield potential [3]. Therefore, investigating the mechanism of drought response at maize seedling stage is very helpful to breed drought-tolerant maize varieties.

Drought response of plants involves gene expression, hormone signaling, ionic equilibrium, metabolite changes, and other aspects [4,5,6]. Drought stress always damages photosystem, reduces photosynthetic capacity, affects carbon fixation, and produces excessive reactive oxygen species (ROS). The content of osmotic regulators and the activity of relevant enzymes in ROS scavenging system often increase correspondingly, so as to maintain ROS homeostasis and reduce the damage of cell membrane system caused by ROS [7,8,9,10,11]. Drought stress also causes seed germination delay, hinders plant growth, shortens flowering period, and leads to insufficient nutrient accumulation in seeds, which ultimately results in a reduction of maize production [12,13,14]. Unfortunately, our understanding of drought response mechanisms is still unclear in maize seedlings.

In recent years, structural and functional genomics, transcriptomics, proteomics, and other omics methods have been wildly used to study molecular mechanisms of drought tolerance in plants. Such studies always accurately and efficiently detected the expression of all genes or the contents of proteins and metabolites in a specific tissue and organ of plants under drought conditions. Proteins are very important for plant stress responding because they are directly involved in plant cell composition and metabolism [15,16]. Therefore, proteomic study can provide new insights to dissect drought response mechanisms at the protein level. High-throughput plant proteomics has been developing rapidly. The isobaric tags for relative and absolute quantification (iTRAQ) analysis method is a second-generation proteomic technique that has been widely used in plant stress response studies [17]. However, just limited studies were recently reported on maize drought response by iTRAQ, in which drought-tolerant and drought-sensitive maize varieties were selected to compare protein profiles under the drought conditions [3,18,19]. Usually, comparative proteomics analysis is an effective strategy to identify pivotal functional proteins and pathways, but it becomes very difficult in maize because of the great differences in genetic background among maize varieties. Consequently, investigating the proteomic changes of each important inbred line should be the first step to investigate the mechanisms of drought tolerance in maize.

Here, an iTRAQ-based quantitative strategy is employed to compare proteome profiles of an important maize inbred line, B73, under the well-watered and water-withheld conditions. The purpose of this study is to summarize the essential proteins and metabolic pathways involved in drought response and to speculate drought resistance strategies of maize seedlings. 

## 2. Results

### 2.1. Phenotypic and Physiological Changes of Maize Seedlings in Response to Drought Stress

**Figure 1:**
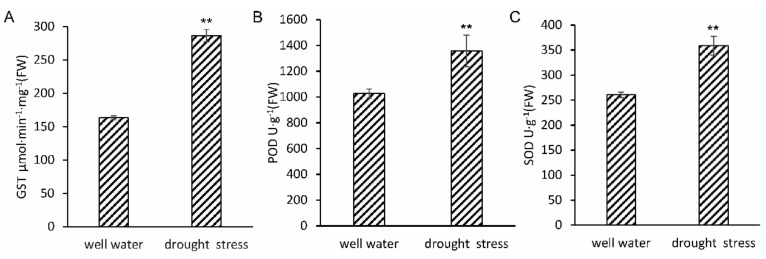
Analysis of GST (**A**), POD (**B**), and SOD (**C**) activities in maize seedlings under drought stress. Data are the means ± SD calculated from four replicates. Statistical significance was determined by a two-sided *t*-test: * *p* < 0.05 and ** *p* < 0.01.

To validate a sampling time point and to investigate the response to drought stress in B73 seedlings, plants at 3-leaf stage were withheld water or not for 5 days in controlled conditions. Three days later, leaf relative water content (RWC) showed significant differences between control and drought treated seedlings, whilst no obviously phenotypic differences were observed, and soil moisture had decreased from ~52% to ~16% under the drought treatment (Table S1). Five days later, 3rd and 4th leaves of well-watered seedlings were obviously longer than that of water-withheld seedlings (Table S2). The activities of peroxidase (POD), superoxide dismutase (SOD), and glutathione S-transferase (GST) were also induced significantly increasing after 5-day water withholding treatment (Figure 1). These results indicated that ROS scavenging system had been activated to maintain ROS homeostasis. Therefore, comparing the proteome differences between the treated and untreated seedlings at this time point, we can explore the early response mechanism of drought tolerance in maize seedling stage. Subsequent results also showed that the abundance of POD (C4J6E4, A5H453, B4FN24, B4FLE3), SOD (P23346), and GST (B6SMJ6, A0A1D6PD99, A0A1D6JYM2, Q9FQA3, A0A1D6LSN2, A0A1D6L6U6) all significantly increased under the drought treatment, indicating that the accumulation of POD, SOD, and GST may be a major reason for the enhancement of ROS scavenging.

### 2.2. Identification of Differentially Accumulated Protein Species (DAPS) by iTRAQ 

A total of 4504 proteins were identified by Paragon, and 3676 trusted proteins were screened based on the criteria described in Section 5. Compared with the well-watered group, the abundance of 2533 proteins in the drought treated group increased by 1.115 times on average, and the abundance of 1758 proteins decreased by an average of 0.911 times. According to the criteria that a protein species was considered differentially accumulated as it exhibited a fold change >1.3 and a *p*-value < 0.05 (*t*-test) with a false discovery rate (FDR) of <1%, 207 DAPS were identified, of which 111 were up-regulated with an average increase of 1.493 times, and 96 were down-regulated with an average decrease of 0.699 times (Table S3). In all DAPS, the most accumulated protein was maize dehydrin DHN1 (A3KLI1) under the drought stress, which was 4.308 times that of the well-watered group; the most significant decrease in protein abundance was the translation initiation factor TAB2 (B4FTR7) involved in the formation of photosystem I (PS I), which was only 0.449 times that of the well-watered group.

### 2.3. Bioinformatics Analysis of DAPS Identified by iTRAQ

DAPS were classified according to the GO functional categories of biological process, molecular function, and cell component. As shown in Table 1, DAPS induced by drought stress in maize seedlings involved in various functional groups, of which biological processes accounted for 15 GO terms (the most representative was “oxidation-reduction process”), molecular functions accounted for 15 GO terms (the most representative was “oxidoreductase activity”), and cellular components accounted for 27 GO terms (the most representative was “cytoplasm”). 

According to GO analysis of biological processes, DAPS significantly enriched in the biological process of photosynthesis and photorespiration, energy metabolism, and carbon fixation. Some DAPS related to plant abiotic stress response, such as regulation of protein stability, oxidation-reduction, and water response, were also significantly changed. Although “oxidation-reduction process” (GO: 0055114, *p* = 8.93 × 10^−3^) was the most representative in all biological process, in which 19 DASP were enriched, the most significantly differential process was “photosynthesis” (GO: 0015979, *p* = 3.49 × 10^−6^). In the process of “response to water” (GO: 0009415), all enriched proteins were dehydrated proteins, and they all significantly accumulated under drought stress, especially DHN1 (A3KLI1), the most up-regulated in all DAPS. GO analysis of molecular functions showed DAPS were classified into 15 categories, in which “oxidoreductase activity” (GO: 0016491, *p* = 5.19 × 10^−3^) was the most representative. “Ribulose-bisphosphate carboxylase activity” was most significant differential function category, in which three DAPS (A0A096TUU6, P05348, and O24574) were down-accumulated. GO analysis of cell components showed that most of the drought-induced DAPS were located in chloroplasts, mitochondria, and related membrane systems. The results of GO analyses showed that photosynthesis was most affected by drought stress in maize seedlings. KEGG analysis also showed that photosynthesis (Pathway ID: zma00195, *p* = 4.06 × 10^−8^) was the most significant enriched pathway, in which eight DAPS, PsaE (B6TH55), PsaL (B6STG2), PsaA (P04966), PsaG (B6U534), PsbH (P24993), PsaG/K (B4G1K9), FNR (B4FI05), and Fd (B4FYW4), were enriched. Except for PsaA (P04966), other DAPS were up-regulated under the drought stress (Figure S1). All these results indicated that drought stress significantly affected photosynthesis in maize seedlings.

### 2.4. qRT PCR Verification

In order to clarify the correspondence between mRNA transcription and protein expression, as well as confirm the authenticity of the iTRAQ analysis, qRT-PCR analysis of 17 protein species were performed. We selected the genes from the 10 categories of DAPS based on the functional annotation (Table 2 and Table 3). Meanwhile, the selected genes should be highly differentiated in response to drought stress and reported to be potentially associated with drought tolerance. The results showed that 15 genes showed the same change tendency on the RNA level as the changes of the corresponding protein abundance, such as gibberellin receptor GID1, dehydrin DHN1, and superoxide dismutase. Besides, two genes of the 60S ribosomal protein and ABC transporter B family member 28 showed opposite trends to the abundance of their corresponding proteins (Table 2). The discrepancy between the transcription level and the abundance of the corresponding protein species probably resulted from various post-translational modification and post-translational modification under the drought stress, such as protein phosphorylation and glycosylation.

## 3. Discussion

Plants have developed various strategies in response to drought stress, including drought escape via a short life cycle or developmental plasticity, drought avoidance via enhanced water uptake and reduced water loss, and drought tolerance via osmotic adjustment, antioxidant capacity, and desiccation tolerance [20]. Proteins are substance basis of life activities and directly involve in plant stress responses. Therefore, proteomics is a new suitable tool for the comprehensive identification of drought-responsive proteins in plants [21,22]. A large number of plant drought resistance related proteins have been identified, which have high potential for crop breeding [3,23,24,25,26]. The integrative analysis of physiological, molecular, and proteomic data most probably provides new clues for further understanding the drought resistance in maize seedlings.

In order to explore the maize seedling responds to drought stress, a proteomics analysis was performed using iTRAQ technique. As a result, a total of 207 DAPS was identified. Due to the problems of repeated statistics and concept ambiguity in the results of GO and KEGG analysis, we performed a more detailed classification analysis of 142 DAPS with accurate functional annotations (Table 3). These DAPS were classified into 10 categories: signaling (7 DAPS), osmotic regulation (5 DAPS), protein synthesis and turnover (31 DAPS), ROS scavenging (14 DAPS), membrane trafficking (19 DAPS), transcription related (19 DAPS), cell structure and cell cycle (9 DAPS), fatty acid metabolism (3 DAPS), carbohydrate and energy metabolism (15 DAPS), and photosynthesis and photorespiration (20 DAPS). 

ASR (ABA-, stress-, and ripening-induced) and CBL (Calcineurin B-like) are two important proteins involved in ABA signaling. Some studies have shown that *ASR* and *CBL* genes up-regulated rapidly when plants were under drought, cold, salt, and weak light stress [27,28]. In this study, the abundance of ASR2 (B4FKG5), ASR3 (A0A1D6EB22), and CBL(B4F9B4) increased significantly under the drought stress, which indicate that ABA signaling is involved in maize seedling drought response. In addition, we found that GID1(K7U051) protein abundance decreased significantly. The function of GID1 is to bind to GA in plants to induce the degradation of DELLAs that inhibit plant growth [29,30,31,32]. Slowing of leaf growth (Table S2) might be partly regulated through GA-GID1-DELLA mediated signaling. We presumed that GA signaling should be an important hormone signal pathway involved in the drought response of maize seedlings. 

Thirty-one DAPS were involved in protein synthesis and turnover, which indicated that this process is very influential in response to drought stress in maize seedlings. The DAPS related to protein synthesis mainly included various ribosomal proteins, translation initiation factor, and peptide chain release factor. The abundance of these proteins all decreased under the drought stress (Table 3). This meant that protein synthesis was weakening in maize seedlings, which may cause slowing down of plant growth and decreasing of water consumption as found in *Phaseolus vulgaris* [33]. As described above, GID1 abundance decreasing may lead to growth inhibition mediated by GA-GID1-DELLA signaling. We inferred that there should be multiplex cross-talk between GA signaling and protein synthesis. Meanwhile, most up-regulated DAPS related to protein turnover were heat shock proteins and chaperones (Table 3), which were essential components for maintaining protein stability and repairing damaged proteins [34,35,36]. The results indicated that the integrity of protein structure was necessary for maize seedlings to enhance drought tolerance. 

Drought stress rapidly lowers the cell division rate in plant leaves [37]. The abundance of several proteins involved in cell structure and cell division were also decreased in this proteomic study, such as a cytoskeleton protein (tubulin: Q41785) and a cell proliferation related protein (nitrilase: B4FQE2). It has been demonstrated that inhibition of Nitrilase expression suppressed Arabidopsis growth and consequently avoided the effects of drought stress [38]. These results suggest that there may also be a mechanism similar to that in Arabidopsis in coping with drought stress by suppressing cell growth. Besides, the abundant of apoptosis related proteins, such as programmed cell death protein 5 (B4FN06) and CASP-like protein cysteinyl aspartate specific proteinase Caspase (A0A1D6QU75), were increased. Transcriptomic and proteomic studies have revealed that up-regulation of programmed apoptosis-related genes is important for maize drought tolerance [2,39,40].

Transcription related proteins are crucial for plants to cope with drought stress. Nineteen DAPS involved in transcription regulation were identified in this study. Chromatin structure modification is a prerequisite to regulate gene transcription. Histones are the major proteins of chromatin and can regulate gene expression [41]. The abundance of histones, especially histone H1, was significantly changed under drought conditions in some plants [12,40,42,43,44]. In addition, some studies have also shown that histone deacetylation promoted gene expression and further influenced the morphology, development, and stress tolerance of plants [45,46,47,48,49]. In this study, we not only identified the abundance of histone H1 (A0A1D6NW49, B4FD93) increased significantly under the drought stress, but also identified the abundance of histone deacetylase (B4F939) decreased. These results indicate that the increase of histone content and the decrease of histone deacetylation in maize seedlings is a means of inhibiting the expression of some genes under drought stress. Unfortunately, the target genes regulated by this means are still unclear.

In this study, all DAPS in osmotic regulation were related to water regulation and up-regulated significantly, in which four DAPS were annotated to dehydrin (DHN) (Table 3). DNH is a class of hydrophilic proteins widely existing in plants. Under various abiotic stress conditions, DHN accumulates rapidly and plays an important role in stabilizing cell membranes and scavenging free radicals [50]. In addition, phosphorylated DHN binds calcium ions to perform the function of molecular chaperone under drought stress [12]. Therefore, the accumulation of DHN (B6SIK2, B4G1H1, A3KLI1, C4J477) suggests that DHN may play an important role in maintaining stable membrane structure, promoting protein synthesis and turnover, as well as ROS scavenging, while the detailed mechanism in drought response of maize seedlings needs to be further studied.

Abiotic stress can stimulate plants to produce excessive ROS and break ROS homeostasis. Therefore, ROS scavenging is very important to improve plant tolerance to abiotic stress. The known ROS scavenging pathways in plants include SOD, POD, and CAT pathways, the ascorbate-glutathione pathway, and the glutathione peroxidase/glutathione s-transferase (GPX/GST) pathway [7,8,9,10,11]. In this study, all identified DAPS involved in ROS scavenging were accumulated. The accumulation of SOD, POD, and GST was consistent with activity increasing of them (Figure 1). According to the different ROS scavenging mechanisms, these DAPS can be classified into two groups. One was antioxidant enzymes, including SOD (P23346) and POD (C4J6E4, A5H453, B4FN24); the other was chemical antioxidant related proteins, including dehydro ascorbate reductase (DHAR: B4FT31), and glutathione s-transferase (GST: B6SMJ6, A0A1D6PD99, A0A1D6JYM2). These proteins should perform similar functions which have been reported in Arabidopsis, rice, wheat, and other plants [40,51,52]. ROS homeostasis is also necessary for maize seedlings to enhance drought tolerance.

In this study, the DAPS classified in membrane trafficking were mostly located in mitochondrial, plasma, or vacuole membranes. Although many membrane proteins have been studied in soybean, wheat, barley, cucumber, and other plants [22,53,54,55], no clear regulatory mechanism was concluded that membrane proteins participated in drought responding process. Moreover, three DAPS related to the fatty acid metabolism were identified in this study. Esterase (B6TZ91) and GDSL esterase (A0A1D6HJU1) were up-regulated under drought stress, while fatty acid export 3 (B4G272) was down-regulated. Based on limited reports, differential expression of fatty acid metabolism-related proteins probably helped to maintain cell membrane integrity and stability under drought stress [56]. The DAPS related cell membrane could be used as reference data for investigating changes of membrane structure and function during drought stress in maize.

Inhibition of photosynthesis is another major influence of drought stress on plants. Recovering photosynthesis is a strategy for plants to cope with drought stress. In this study, the most of drought-increased DAPS involved in photoreactions, which suggested photosynthesis in maize seedlings was maintained under the water-deficient condition. For example, the expression of cytochrome b related proteins were also significantly up-regulated in response to drought in Arabidopsis [57] and apples [13]. Unexpectedly, TAB2 (B4FTR7), a regulatory protein related to PS I assembly, was significantly down-regulated under drought stress and then probably inhibited photosynthesis, which contradicted with the abundance increasing of other photosynthesis related DAPS identified in this study. Some studies verified that TAB2 was closely related to the transcriptional regulation of glycolytic enzymes [58,59]. These results indicated that TAB2 might be involved in complex interactions between photosynthesis and glucose metabolism under drought stress.

## 4. Materials and Methods

### 4.1. Plant Materials and Drought Stress Treatments

Maize inbred line B73 was used in this experiment because of its abundant database resources and important breeding value. Seeds were surface sterilized with 70% alcohol for 1 min and then 3% NaClO for 10 min, thoroughly rinsed with distilled water and germinated on filter paper wetted with distilled water in plates at 26 °C for 3 days. Germinated seeds were transplanted into pots containing 200 g fully dried soil, and then the water content of soil was maintained at 50%. The seedlings were grown under controlled conditions (light/dark cycles: 14h/10h; light intensity: 70 mmol/m^2^s; temperature: 28/22 °C; relative humidity: 60% ± 5%) to 3-leaf stage. Then, a half of seedlings were exposed to drought stress that water was withheld from the seedlings for 5 days, while the rest seedlings were still grown under the well-watered conditions. Leaf samples were collected after 0, 1, 3, and 5 days of treatment (for measuring leaf length and leaf relative water content (RWC)). On the fifth day after water withdrawing treatment, all green tissues from every 5 individual plants were mixed as one biological replicate to be stored in liquid nitrogen. Four biological replicates were respectively collected from the treatment and control groups for protein extraction and subsequent analysis 

### 4.2. iTRAQ Analysis

iTRAQ analysis was carried out by Shanghai Luming Biotechnology Co., LTD. The standard iTRAQ analysis was performed with minor modifications as previously described [60,61] briefly, including protein preparation, iTRAQ labeling and SCX fractionation, LC-ESI-MS/MS analysis, protein identification and data analysis, and bioinformatics analysis.

Data was processed with Protein Pilot Software v. 5.0 (AB SCIEX, USA) against the Uniprot database (available online: https://www.uniprot.org; accessed on 12 January 2018; uniprot_*Zea mays_*132339_20180112.FASTA; 76,417 sequences) using the Paragon algorithm [62]. The experimental data from tandem mass spectrometry (MS) was used to match the theory data to obtain result of protein identification. Protein identification was performed with the search option: emphasis on biological modifications.

To reduce the probability of false peptide identification, only peptides with significance scores (≥20) at the 95% confidence interval by a Paragon probability analysis greater than “identity” were counted as identified. Each confident protein identification involves at least one unique peptide. For protein quantitation, it was required that a protein contains at least two unique peptides. The quantitative protein ratios were weighted and normalized by the median ratio in Paragon. We only used ratios with *p*-values < 0.05 (*t*-test), and only fold changes of >1.3 were considered as significant on the basis of the related iTRAQ studies [63,64,65].

Functional annotations of differentially accumulated protein species were performed using Gene Ontology (GO) (http://www.geneontology.org). The Kyoto Encyclopedia of Genes and Genomes (KEGG) (http://www.genome.jp/kegg) was used to predict the main metabolic pathways and biochemical signals transduction pathways that involved the DAPS. A *p*-value <0.05 (Fisher’s exact test) was used as the threshold to determine the significant enrichments of GO and KEGG pathways.

### 4.3. qRT PCR Verification

Total RNA was isolated using RNAiso Plus reagent (TaKaRa) from no less than 3 seedlings. To remove genomic DNA contamination, total RNA was treated with the TURBO DNA-free™ Kit (Ambion). The concentration of total RNA was determined using a Nanodrop2000c (Thermo Scientific, USA). One microgram of total RNA was used to synthesize cDNA with the TransScript All-in-One First-Strand cDNA Synthesis SuperMix for qPCR kit (Transgen Biotech). To validate the differentially accumulated protein obtained from iTRAQ, 17 genes (Table 2) were subjected to quantitative real-time PCR. All reactions were performed in triplicate, including the non-template controls. Data were quantified using the comparative CT method (2^−ΔΔCT^ method) [66].

### 4.4. Antioxidants Assays

The activities of POD, SOD, and GST in shoots were respectively assayed using detection kits (POD-1-Y, SOD-1-Y, and GST-2-W) from Suzhou Comin Biotechnology Co. Ltd., following the manufacturer’s instructions. Statistical data were obtained from four independent experiments. All values were the means of four assays carried out for each value. Data analysis was performed using the SPSS statistical software package (version 19.0; SPSS Institute Ltd., Armonk, NY, USA), and the significance of differences were tested by *t*-test with a *p* values < 0.05 set as statistically significant.

## 5. Conclusions

**Figure 2:**
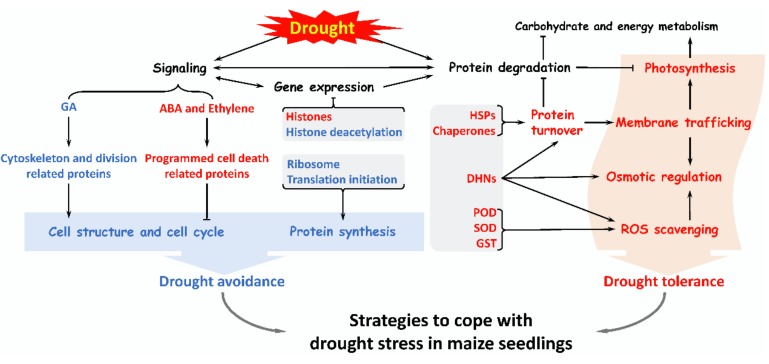
A summary of various pathways in maize seedlings in response to drought stress. Drought stress activates several signaling to regulates some gene expression, and enhances ROS scavenging, osmotic regulation, protein turnover, membrane trafficking, and photosynthesis, which improves drought tolerance of maize seedlings. Besides, drought stress inhibits some protein synthesis as well as the cytoskeleton and cell division to avoid excessive water loss. Importantly, maize seedlings enable a complex set of strategies to cope with drought stress.

Proteomics is a powerful tool to analyze the mechanisms of drought response and tolerance in maize which hardly revealed by transcriptomic or genomic technologies. However, the studies on maize proteomes related with drought response are still very limited. In this study, we found that more than 200 DAPS were drought-responsive in maize seedlings, which were involved in drought signal transduction, ROS scavenging, osmotic regulation, specific gene expression regulation, protein synthesis and turnover, cell structure modulation, as well as other metabolisms. A hypothetical strategy was proposed that maize seedlings coped with drought stress by improving drought tolerance (via promoting osmotic adjustment and antioxidant capacity, maintaining membrane integrity and stability, as well as recovering photosynthesis) and enhancing drought avoidance (via inhibiting cell division and protein synthesis to reduce water loss) (Figure 2). All these findings enrich the proteome data of maize. The DAPS will provide candidate genes/proteins for genetic improvement in maize drought tolerance. In the future, the integration of genomics, proteomics, transcriptomics, and metabolomics will help us to understand the drought response mechanism of maize seedlings.

## Figures and Tables

**Table 1:** Gene Ontology (GO) annotation of drought-responsive differentially accumulated protein species (DAPS) in maize seedlings.

	GO_Name	GO_ID	*p*-Value	Count	Differentially Accumulated Protein Species (DAPS) ^1^
**Biological Process**					
1	photosynthesis	GO:0015979	3.49 × 10^−6^	9	B6TH55|1.36818125; P05348|0.71520625; B6STG2|1.30215625; O24574|0.6964125; P04966|0.76561875; B6U534|1.40439375; P24993|1.39320625; B4G1K9|1.3451375; B4FTR7|0.448975
2	mitochondrial electron transport	GO:0006122	6.51 × 10^−5^	3	B6TEX6|1.324725; B6SPA1|1.36054375; Q6R9D5|1.34895
3	carbon fixation	GO:0015977	6.51 × 10^−5^	3	A0A096TUU6|0.55751875; P05348|0.71520625; O24574|0.6964125
4	photorespiration	GO:0009853	1.86 × 10^−4^	3	P05348|0.71520625; O24574|0.6964125; B6TVC7|1.37949375
5	ATP synthesis coupled electron transport	GO:0042773	6.03 × 10^−4^	3	B6TEX6|1.324725; B6SPA1|1.36054375; Q6R9D5|1.34895
6	response to water	GO:0009415	9.14 × 10^−4^	3	C4J477|2.24320625; B4G1H1|2.28670625; A3KLI1|4.3088125
7	mitochondrial transmembrane transport	GO:1990542	1.12 × 10^−3^	2	B6U5I0|1.344925; B4FET7|0.765575
8	respiratory electron transport chain	GO:0022904	1.19 × 10^−3^	3	B6TEX6|1.324725; B6SPA1|1.36054375; Q6R9D5|1.34895
9	electron transport chain	GO:0022900	1.50 × 10^−3^	4	B6TEX6|1.324725; B6SPA1|1.36054375; B4FYW4|1.45836875; Q6R9D5|1.34895
10	cellular metabolic compound salvage	GO:0043094	3.21 × 10^−3^	3	P05348|0.71520625; O24574|0.6964125; B6TVC7|1.37949375
11	response to inorganic substance	GO:0010035	3.50 × 10^−3^	4	C4J477|2.24320625; B4G1H1|2.28670625; P23346|1.48615; A3KLI1|4.3088125
12	seed maturation	GO:0010431	6.23 × 10^−3^	2	B6UH30|1.92453125; C0PGB5|0.717475
13	regulation of protein stability	GO:0031647	7.15 × 10^−3^	1	P24993|1.39320625
14	S-glycoside biosynthetic process	GO:0016144	7.79 × 10^−3^	2	B4FTR7|0.448975; B4FJN0|0.654025
15	oxidation-reduction process	GO:0055114	8.93 × 10^−3^	19	B4FRC8|1.38471875; P05348|0.71520625; B4F8L7|0.6962375; B4FT31|1.31011875; A5H453|1.357525; B6THA1|1.336; O24574|0.6964125; P04966|0.76561875; B6TEX6|1.324725; K7W7R1|1.62785625; B4FN24|1.3030875; K7VH40|1.4048; B6SPA1|1.36054375; P23346|1.48615; B4FI05|1.33465625; C4J6E4|1.34324375; Q41738|0.7225625; B4FYW4|1.45836875; Q6R9D5|1.34895
**Molecular Function**					
1	ribulose-bisphosphate carboxylase activity	GO:0016984	2.43 × 10^−6^	3	A0A096TUU6|0.55751875; P05348|0.71520625; O24574|0.6964125
2	rRNA binding	GO:0019843	8.14 × 10^−5^	4	C4JBF5|0.72338125; P08527|0.73171875; P06586|0.7672125; B6SX84|0.759125
3	electron carrier activity	GO:0009055	2.76 × 10^−4^	6	B6THA1|1.336; P04966|0.76561875; B6TVC7|1.37949375; B4FYW4|1.45836875; Q6R9D5|1.34895; K7V5H2|0.76678125
4	carboxy-lyase activity	GO:0016831	6.22 × 10^−4^	4	A0A096TUU6|0.55751875; AY110562|1.3718125; P05348|0.71520625; O24574|0.6964125
5	chlorophyll binding	GO:0016168	7.99 × 10^−4^	3	P04966|0.76561875; B6U534|1.40439375; B4G1K9|1.3451375
6	ubiquinol-cytochrome-c reductase activity	GO:0008121	1.94 × 10^−3^	2	B6SPA1|1.36054375; Q6R9D5|1.34895
7	phosphate ion binding	GO:0042301	3.12 × 10^−3^	1	P24993|1.39320625
8	structural molecule activity	GO:0005198	3.81 × 10^−3^	9	C4JBF5|0.72338125; Q41785|0.6788375; P08527|0.73171875; B6SJ08|0.7401; P06586|0.7672125; B6SJU8|0.7215; B6SX84|0.759125; B6SIT5|0.6876; B6T2K5|0.73715
9	oxidoreductase activity	GO:0016491	5.19 × 10^−3^	17	B4FRC8|1.38471875; P05348|0.71520625; B4F8L7|0.6962375; B4FT31|1.31011875; A5H453|1.357525; B6THA1|1.336; O24574|0.6964125; P04966|0.76561875; K7W7R1|1.62785625; B4FN24|1.3030875; K7VH40|1.4048; P23346|1.48615; B6SPA1|1.36054375; B4FI05|1.33465625; C4J6E4|1.34324375; Q41738|0.7225625; Q6R9D5|1.34895
10	structural constituent of ribosome	GO:0003735	5.57 × 10^−3^	8	C4JBF5|0.72338125; P08527|0.73171875; B6SJ08|0.7401; P06586|0.7672125; B6SJU8|0.7215; B6SX84|0.759125; B6SIT5|0.6876; B6T2K5|0.73715
11	arginine decarboxylase activity	GO:0008792	6.23 × 10^−3^	1	AY110562|1.3718125
12	myristoyltransferase activity	GO:0019107	6.23 × 10^−3^	1	Q4FZ48|0.70004375
13	lyase activity	GO:0016829	7.00 × 10^−3^	6	A0A096TUU6|0.55751875; AY110562|1.3718125; A0A096RZN2|0.6292; P05348|0.71520625; O24574|0.6964125; B4FJJ9|0.75581875
14	ferredoxin-NADP+ reductase activity	GO:0004324	9.34 × 10^−3^	1	B4FI05|1.33465625
15	hydrolase activity, acting on ether bonds	GO:0016801	9.34 × 10^−3^	1	C0PHR4|0.74545
**Cell Component**					
1	cytoplasm	GO:0005737	1.32 × 10^−9^	35	C4J030|0.6855625; Q9ZT00|0.6089125; P05348|0.71520625; C4JBF5|0.72338125; B6SLX1|1.35598125; P23346|1.48615; B6TEX6|1.324725; Q41785|0.6788375; O24574|0.6964125; P04966|0.76561875; J7LC26|0.76554375; Q6R9D5|1.34895; P24993|1.39320625; P08527|0.73171875; B6SJ08|0.7401; P06586|0.7672125; B6SPA1|1.36054375; B6U5I0|1.344925; B6SJU8|0.7215; B6TVC7|1.37949375; B4FET7|0.765575; K7U772|0.73978125; B4FTR7|0.448975; B4FJN0|0.654025; B6SX84|0.759125; B4F8V9|0.74715; Q41738|0.7225625; B6SIT5|0.6876; B6SNM4|1.3185375; K7V2H9|1.3191875; K7W104|1.4398125; B6SLJ2|1.339075; C4JBA7|0.7688625; B6U1I6|1.69764375; B6T2K5|0.73715
2	chloroplast	GO:0009507	1.49 × 10^−7^	14	C4J030|0.6855625; Q9ZT00|0.6089125; P05348|0.71520625; O24574|0.6964125; P04966|0.76561875; B4FTR7|0.448975; P24993|1.39320625; P06586|0.7672125; K7U772|0.73978125; B4FJN0|0.654025; B6SX84|0.759125; Q41738|0.7225625; P08527|0.73171875; B6TVC7|1.37949375
3	macromolecular complex	GO:0032991	1.10 × 10^−6^	25	B6TH55|1.36818125; C4JBF5|0.72338125; B6U471|0.7354625; Q41785|0.6788375; B6SX84|0.759125; Q6R9D5|1.34895; B4FRU4|1.4294875; P04966|0.76561875; B6U534|1.40439375; B6TEX6|1.324725; J7LC26|0.76554375; B6SIT5|0.6876; P24993|1.39320625; P08527|0.73171875; B4G1K9|1.3451375; B6SJ08|0.7401; P06586|0.7672125; B4FD93|1.6656125; B6SJU8|0.7215; B4FET7|0.765575; B6SNM4|1.3185375; K7V2H9|1.3191875; B4FJ31|1.3158875; B6STG2|1.30215625; B6T2K5|0.73715
4	membrane protein complex	GO:0098796	1.10 × 10^−6^	11	B6TH55|1.36818125; P04966|0.76561875; B6TEX6|1.324725; B4FET7|0.765575; Q6R9D5|1.34895; P24993|1.39320625; B4G1K9|1.3451375; B6U534|1.40439375; B6SNM4|1.3185375; K7V2H9|1.3191875; B6STG2|1.30215625
5	photosystem I	GO:0009522	1.30 × 10^−6^	5	B6TH55|1.36818125; B6STG2|1.30215625; P04966|0.76561875; B6U534|1.40439375; B4G1K9|1.3451375
6	mitochondrial inner membrane	GO:0005743	1.38 × 10^−6^	6	B6TEX6|1.324725; B6SPA1|1.36054375; B4FET7|0.765575; K7V2H9|1.3191875; K7W104|1.4398125; Q6R9D5|1.34895
7	mitochondrion	GO:0005739	9.15 × 10^−6^	10	B6TEX6|1.324725; B6SPA1|1.36054375; B6U5I0|1.344925; B6TVC7|1.37949375; B4FET7|0.765575; Q6R9D5|1.34895; K7U772|0.73978125; K7V2H9|1.3191875; K7W104|1.4398125; B6SLJ2|1.339075
8	photosystem	GO:0009521	9.21 × 10^−6^	6	B6TH55|1.36818125; B6STG2|1.30215625; P04966|0.76561875; B6U534|1.40439375; P24993|1.39320625; B4G1K9|1.3451375
9	respiratory chain	GO:0070469	4.46 × 10^−5^	4	B6TEX6|1.324725; B6SPA1|1.36054375; K7V2H9|1.3191875; Q6R9D5|1.34895
10	respiratory chain complex	GO:0098803	5.15 × 10^−5^	3	B6TEX6|1.324725; K7V2H9|1.3191875; Q6R9D5|1.34895
11	organelle envelope	GO:0031967	1.96 × 10^−4^	7	C4J030|0.6855625; B6TEX6|1.324725; B6SPA1|1.36054375; B4FET7|0.765575; K7V2H9|1.3191875; Q6R9D5|1.34895; K7W104|1.4398125
12	photosynthetic membrane	GO:0034357	2.71 × 10^−4^	6	B6TH55|1.36818125; B6STG2|1.30215625; P04966|0.76561875; B6U534|1.40439375; P24993|1.39320625; B4G1K9|1.3451375
13	respiratory chain complex III	GO:0045275	4.39 × 10^−4^	2	B6TEX6|1.324725; Q6R9D5|1.34895
14	mitochondrial protein complex	GO:0098798	6.73 × 10^−4^	3	B6TEX6|1.324725; B4FET7|0.765575; K7V2H9|1.3191875
15	ribonucleoprotein complex	GO:0030529	7.42 × 10^−4^	11	C4JBF5|0.72338125; B6U471|0.7354625; J7LC26|0.76554375; P08527|0.73171875; B6SJ08|0.7401; P06586|0.7672125; B6SJU8|0.7215; B6SX84|0.759125; B6SIT5|0.6876; B4FJ31|1.3158875; B6T2K5|0.73715
16	thylakoid	GO:0009579	7.84 × 10^−4^	6	B6TH55|1.36818125; B6STG2|1.30215625; P04966|0.76561875; B6U534|1.40439375; P24993|1.39320625; B4G1K9|1.3451375
17	photosystem I reaction center	GO:0009538	8.81 × 10^−4^	2	B6TH55|1.36818125; B6STG2|1.30215625
18	mitochondrial membrane part	GO:0044455	9.16 × 10^−4^	3	B6TEX6|1.324725; B4FET7|0.765575; K7V2H9|1.3191875
19	protein complex	GO:0043234	1.69 × 10^−3^	14	B6TH55|1.36818125; Q41785|0.6788375; P04966|0.76561875; Q6R9D5|1.34895; B4FET7|0.765575; B6TEX6|1.324725; P24993|1.39320625; B4G1K9|1.3451375; B4FD93|1.6656125; B6SNM4|1.3185375; B4FRU4|1.4294875; K7V2H9|1.3191875; B6U534|1.40439375; B6STG2|1.30215625
20	chloroplast part	GO:0044434	1.96 × 10^−3^	6	C4J030|0.6855625; Q9ZT00|0.6089125; P04966|0.76561875; P24993|1.39320625; K7U772|0.73978125; B4FJN0|0.654025
21	oxidoreductase complex	GO:1990204	2.26 × 10^−3^	3	B6TEX6|1.324725; K7V2H9|1.3191875; Q6R9D5|1.34895
22	ribosome	GO:0005840	2.29 × 10^−3^	9	C4JBF5|0.72338125; J7LC26|0.76554375; P08527|0.73171875; B6SJ08|0.7401; P06586|0.7672125; B6SJU8|0.7215; B6SX84|0.759125; B6SIT5|0.6876; B6T2K5|0.73715
23	mitochondrial respiratory chain	GO:0005746	2.40 × 10^−3^	2	B6TEX6|1.324725; K7V2H9|1.3191875
24	chloroplast stroma	GO:0009570	2.51 × 10^−3^	4	C4J030|0.6855625; Q9ZT00|0.6089125; K7U772|0.73978125; B4FJN0|0.654025
25	organelle membrane	GO:0031090	3.43 × 10^−3^	7	B6TEX6|1.324725; B6SPA1|1.36054375; B4FET7|0.765575; B6SNM4|1.3185375; K7V2H9|1.3191875; Q6R9D5|1.34895; K7W104|1.4398125
26	viral nucleocapsid	GO:0019013	3.56 × 10^−3^	2	B6U471|0.7354625; B4FJ31|1.3158875
27	intracellular membrane-bounded organelle	GO:0043231	4.14 × 10^−3^	26	C4J030|0.6855625; Q9ZT00|0.6089125; P05348|0.71520625; O24574|0.6964125; P04966|0.76561875; B6TEX6|1.324725; P24993|1.39320625; P06586|0.7672125; B6SPA1|1.36054375; B4FD93|1.6656125; Q6R9D5|1.34895; B6U5I0|1.344925; B6TVC7|1.37949375; B4FET7|0.765575; K7U772|0.73978125; B4FJN0|0.654025; B6SX84|0.759125; Q41738|0.7225625; B6SNM4|1.3185375; K7V2H9|1.3191875; K7W104|1.4398125; B6SLJ2|1.339075; B4FTR7|0.448975; B4FJ31|1.3158875; P08527|0.73171875; B6U1I6|1.69764375

^1^ Before “|” is Uniprot accession number of DAPS, after “|” is DAPS fold change, which is expressed as the ratio of intensities of up-regulated or down-regulated proteins between drought stress treatments and control (well-watered conditions).

**Table 2:** Quantitative real-time PCR (qRT-PCR) information of the selected genes encoding DAPS.

Accession No.		Fold Change ^1^		qRT-PCR Primers	Protein Description
UniProt	B73 RefGen_v4	iTRAQ	qRT-PCR		
B4FKG5	Zm00001d023529	2.02 ± 0.28	2.73 ± 0.32	F: ACCACCTGTTCCACCACAAG R: CTCCTCCTCGATCTTGTGGC	Abscisic acid stress ripening protein 2
K7U051	Zm00001d050493	0.68 ± 0.11	0.23 ± 0.11	F: GACTTCTCCCGCCTCTACCT R: CGGTTGAGGAAGTCGCTGAT	Gibberellin receptor GID1L2
A3KLI1	Zm00001d037894	4.31 ± 0.34	7.44 ± 0.49	F: CGCGTCAAAGCCGTAATGTT R: TGAACAGTACACGGACCCAG	Dehydrin DHN1
B6T2K5	Zm00001d004052	0.74 ± 0.19	1.15 ± 0.09	F: TTCATATCCTCACTCGCCGC R: CGCTTCTTTTCCCTCTCGGT	60S ribosomal protein L35
C4J410	Zm00001d012420	1.41 ± 0.17	1.97 ± 0.08	F: TCAAGAAGAAGGTGGACGCC R: GTTGCAGATCCCCTCAAGCT	Heat shock protein1
Q9FQA3	Zm00001d020780	1.36 ± 0.23	1.74 ± 0.19	F: CATCGACGAGGTCTGGAAGG R: CCGAACCAGGCCTTCATCAG	Glutathione transferase GST
A5H453	Zm00001d022456	1.36 ± 0.10	1.67 ± 0.05	F: GACATGGTCGCTCTCTCAGG R: CGAGGTTCCCCATCTTCACC	Peroxidase 42
P23346	Zm00001d047479	1.49 ± 0.17	1.50 ± 0.24	F: CCAGAAGATGAGAACCGCCA R: GCCCACCCTTTCCAAGATCA	Superoxide dismutase [Cu-Zn] 4AP
A0A1D6HZB6	Zm00001d019627	0.52 ± 0.19	1.02 ± 0.09	F: GGCTACTAGTGCACTGGACG R: AAGCAAGTCTCTGTGTCCCG	ABC transporter B family member 28
B8A390	Zm00001d046591	0.68 ± 0.14	0.34 ± 0.10	F: GGTGTGCAGAAGACGGTGTA R: CCTTCCCAATGGCAGCAGTA	Vacuolar-type H^+^-pyrophosphatase5
K7TI82	Zm00001d024703	1.32 ± 0.58	2.47 ± 0.08	F: ACGGCGACAAGGGTAAGAAG R: TGTCCACGACCTTCTTCACG	C3H transcription factor
Q41785	Zm00001d040508	0.68 ± 0.22	0.30 ± 0.05	F: TTGTGATATCCCTCCGCGTG R: CGTCCTCATATTCCGCCTCC	Tubulin beta-8 chain O
A0A1D6HJU1	Zm00001d017989	1.32 ± 0.23	2.13 ± 0.15	F: CAGCGTGGTGTCCTACTTCA R: GCTGCTTGAAGTTGATGGGC	GDSL esterase/lipase
B4F8L7	Zm00001d027488	0.70 ± 0.06	0.18 ± 0.13	F: AACACCGTGAAGACTGGCAT R: TCGTACACCTTGCACTCGTC	Glyceraldehyde-3-phosphate dehydrogenase
C0PGB5	Zm00001d043986	0.72 ± 0.21	0.32 ± 0.12	F: CTCCAACCCGAGCAGAAGTT R: CTTTGAACGAGCGCAACCTC	Pyruvate kinase
B6TH55	Zm00001d005446	1.37 ± 0.29	1.44 ± 0.14	F: AGGCGCCAAGGTGAAGATC R: CTCGTCCAAGGCGTAGTTGT	Photosystem I reaction center subunit IV A
B4FTR7	Zm00001d017179	0.45 ± 0.24	0.85 ± 0.02	F: ACTGGAGAGGAGGTACGCAT R: CCGTCGGAGTTGAGGTTCTC	Tab2 protein

^1^ Fold change is expressed as the ratio of intensities of up-regulated or down-regulated proteins/genes between drought stress treatments and control (well-watered conditions); All the fold change figures below 1 or above 1 represent that the genes were down-regulated or up-regulated, respectively.

**Table 3:** Classification of drought-responsive DAPS with detailed annotation.

Accession No.^1^	Sequence Coverage (%) ^2^	Peptides (95%) ^3^	Fold Change ^4^	Description
**Signaling**				
A0A1D6EB22	61.3	39	1.76909375	Abscisic acid stress ripening3
B4FKG5	73.9	36	2.01644375	Abscisic acid stress ripening protein 2
B6U1I6	11.5	1	1.69764375	B-cell receptor-associated protein 31-like containing protein
B4F9B4	5.6	1	1.452825	Calcineurin B-like protein
K7U3I3	11.3	2	1.3692375	Ethylene response protein
A0A1D6HP31	5.3	1	1.31536875	F-box protein PP2-B10
K7U051	8.6	1	0.68396875	Gibberellin receptor GID1L2
**Osmotic Regulation**				
B6SIK2	24	3	1.44823125	Dehydrin 13
B4G1H1	36.9	9	2.28670625	Dehydrin COR410
A3KLI1	45.2	18	4.3088125	Dehydrin DHN1
C4J477	46.7	20	2.24320625	Dehydrin DHN2
B4G019	45.3	13	1.57125625	Hydroxyproline-rich glycoprotein family protein
**Protein Synthesis and Turnover**				
B6TDB5	22.8	2	1.3013375	17.4 kDa class I heat shock protein 3
B4FRU4	28.5	8	1.4294875	26S proteasome non-ATPase regulatory subunit 4-like protein
P08527	31.1	3	0.73171875	30S ribosomal protein S14, chloroplastic
P06586	29.5	3	0.7672125	30S ribosomal protein S3, chloroplastic
B4FQS5	21.2	4	1.3009375	36.4 kDa proline-rich protein
B6SIT5	62.8	7	0.6876	60S acidic ribosomal protein P2A
B6SJ08	55.6	9	0.7401	60S ribosomal protein L18
B6T2K5	32.5	3	0.73715	60S ribosomal protein L35
C4JBF5	77.4	14	0.72338125	60S ribosomal protein L9
B6SLX1	70.4	10	1.35598125	Chaperonin
Q4FZ48	39.7	2	0.70760625	Cysteine proteinase inhibitor
B6TNU0	16.8	4	0.6811	Eukaryotic translation initiation factor 2 gamma subunit
K7VH40	41.7	7	1.4048	Flavoprotein wrbA
K7U772	7.7	2	0.73978125	Glutamyl-tRNA(Gln) amidotransferase subunit B, chloroplastic/mitochondrial
B4FU07	14.2	1	0.76964375	Glycoprotein membrane GPI-anchored
B4FJN0	8.3	2	0.654025	Phosphoglucan phosphatase DSP4 chloroplastic
C4J410	59.4	59	1.4112625	Heat shock protein1
A0A1D6PTR2	33.2	10	1.58213125	HSP20-like chaperones superfamily protein
B6T903	68.1	34	1.66753125	Nascent polypeptide-associated complex alpha subunit-like protein
A0A1D6L313	3.9	2	1.92525	Peptidase
A0A1D6PP97	12.4	2	0.69133125	Peptide chain release factor APG3 chloroplastic
B6SX84	6.6	1	0.759125	Plastid-specific ribosomal protein 6
B6U471	56.7	34	0.7354625	Ribonucleoprotein A
B6SJU8	19.1	2	0.7215	Ribosomal protein L15
J7LC26	40.8	7	0.76554375	Ribosomal protein S10
A0A096TUU6	63.4	206	0.55751875	Ribulose bisphosphate carboxylase large chain
O24574	79.4	52	0.6964125	Ribulose bisphosphate carboxylase small chain
P05348	79.4	54	0.71520625	Ribulose bisphosphate carboxylase small chain, chloroplastic
Q9ZT00	64.4	42	0.6089125	Ribulose bisphosphate carboxylase/oxygenase activase, chloroplastic
A0A1D6JXL4	14.3	2	0.6313	Serine/threonine-protein phosphatase
B4F8V9	44.1	15	0.74715	T-complex protein 1 subunit delta
**Reactive Oxygen Species (ROS) Scavenging Pathways**				
B4FT31	82.7	14	1.31011875	Dehydro ascorbate reductase
B6SMJ6	3.3	1	1.54051875	Glutathione S-transferase GSTU6
A0A1D6PD99	65.8	22	1.5907375	Glutathione S-transferase L2 chloroplastic
A0A1D6JYM2	36.7	25	1.606675	Glutathione S-transferase L2 chloroplastic
Q9FQA3	12.2	2	1.35623125	Glutathione transferase GST
A0A1D6LSN2	51.6	9	1.58461875	Glutathione transferase19
A0A1D6L6U6	44.2	7	1.3986625	Glutathione transferase5
B6THA1	66.4	8	1.336	Grx_C2.2-glutaredoxin subgroup I
C0PBY7	45.8	13	1.448225	Nucleoside diphosphate kinase
C4J6E4	52	28	1.34324375	Peroxidase
A5H453	44.2	26	1.357525	Peroxidase 42
B4FN24	71	21	1.3030875	Peroxiredoxin-5
B4FLE3	20.5	1	1.36374375	Prostaglandin E synthase 3
P23346	59.2	21	1.48615	Superoxide dismutase [Cu-Zn] 4AP
**Membrane Trafficking**				
A0A1D6HZB6	10.4	1	0.52055	ABC transporter B family member 28 (ATP-Binding Cassette)
A0A1D6P3C1	23.4	7	1.4758	Charged multivesicular body protein 4b
A0A1D6HQA8	12.1	1	0.62341875	Chloroplast channel forming outer membrane protein
K7V5H2	7.1	1	0.76678125	Copper ion binding protein
B6SQL3	83	9	1.339825	Copper transport protein CCH
B6TJH8	12.1	2	1.3348125	Mitochondrial glycoprotein
B6U5I0	47	4	1.344925	Mitochondrial import inner membrane translocase subunit Tim10
B6SLJ2	32.5	1	1.339075	Mitochondrial import inner membrane translocase subunit Tim8
B4FET7	11.8	2	0.765575	Mitochondrial import inner membrane translocase subunit TIM21
A0A1D6JKW8	43.9	4	1.63820625	Non-specific lipid-transfer protein
B6SGP7	67.8	98	1.4372375	Non-specific lipid-transfer protein
B4FB54	74	11	1.51785	Non-specific lipid-transfer protein
B6UH30	42.5	8	1.92453125	PEBP (Phosphatidylethanolamine-binding protein) family protein
B6SNM4	39.3	3	1.3185375	Protein transport protein Sec61 beta subunit
A0A1D6LHK0	38.9	5	1.3837125	Protein transport protein Sec61 subunit beta
A0A1D6PHG2	3.9	1	0.65433125	SecY protein transport family protein
B4FSU1	14.9	2	0.7646625	Transmembrane emp24 domain-containing protein 10
B6SUM0	16.5	1	0.51840625	Vacuolar protein sorting-associated protein 29
B8A390	10.5	4	0.6845	Vacuolar-type H+-pyrophosphatase5
**Transcription Related**				
C0PHR4	58.4	25	0.74545	Adenosyl homocysteinase
A0A1D6HZB8	18.3	3	1.55415	Alkyl transferase
K7TI82	2	1	1.3210625	C3H transcription factor
B6SSH9	57.1	30	1.6191	Extracellular ribonuclease LE OS=Zea mays PE=2 SV=1
A0A1D6J4Q0	30.3	6	0.5743625	Flowering-promoting factor 1-like protein
A0A1D6NGH2	6.1	3	1.82566875	GBF-interacting protein
B4F939	9.6	1	0.7477625	Histone deacetylase
A0A1D6NW49	33.6	3	1.44954375	Histone H1
B4FD93	33.7	5	1.6656125	Histone H1
C0P4Y9	25	2	1.37089375	HNH endonuclease
K7TNM4	32.8	8	1.44975	Nuclear transport factor 2 (NTF2) family protein with RNA binding (RRM-RBD-RNP motifs) domain
A0A1D6E366	17.7	2	1.40421875	Nucleosome/chromatin assembly factor D
A0A1D6HNJ8	17.6	2	0.66309375	Plastid transcriptionally active 17
K7THT7	4.2	1	0.7220125	Putative DEAD-box ATP-dependent RNA helicase family protein
B4FQT5	6.2	1	1.77780625	Replication factor C subunit 2
B4FBD6	12.2	3	1.4390625	Ribonuclease 3
B4FK28	15.1	3	0.7205	RNA-binding (RRM/RBD/RNP motifs) family protein
B4FJ31	20.9	1	1.3158875	Small nuclear ribonucleoprotein F
A0A1D6EC40	6.3	1	0.75345	Trihelix transcription factor ASR3
**Cell Structure and Cell Cycle**				
A0A1D6QU75	4.3	1	1.5890375	CASP-like protein cysteinyl aspartate specific proteinase Caspase
A0A1D6L6F4	6.8	3	0.73449375	Glucose-6-phosphate isomeras
A0A1D6E965	68.5	80	0.71039375	Glyceraldehyde-3-phosphate dehydrogenase
B4FQE2	12.5	1	0.7307125	Nitrilase 4 isoform 1
B4FV91	51.7	10	1.4576125	Pathogenesis related protein5
B4FN06	46.9	5	1.40720625	Programmed cell death protein 5
C0HE67	9.3	1	1.4680625	Protein WVD2-like 5
C0P5V6	19.2	5	0.7639625	Transferase
Q41785	23.6	8	0.6788375	Tubulin beta-8 chain O
**Fatty Acid Metabolism**				
B6TZ91	6.5	1	1.32930625	Esterase
A0A1D6HJU1	19.2	2	1.3182	GDSL esterase/lipase
B4G272	25	14	0.75193125	Protein fatty acid export 3 chloroplastic
**Carbohydrate and Energy Metabolism**				
A0A1D6IZQ8	76.4	10	1.34993125	Acyl-CoA-binding protein1
B4FJJ9	7.5	1	0.75581875	ATP-dependent (S)-NAD(P)H-hydrate dehydratase
B6SWK9	7.2	2	0.66775625	Auxin-induced beta-glucosidase
A0A1D6N5T1	10.2	3	0.76715	Beta-galactosidase
B4FD17	32.2	13	0.67739375	Dihydrolipoamide acetyltransferase component of pyruvate dehydrogenase complex
B6T5U0	39.8	9	1.325275	F1F0-ATPase inhibitor protein
B6TGD4	54.2	8	1.344875	F1F0-ATPase inhibitor protein
B4F8L7	54.8	33	0.6962375	Glyceraldehyde-3-phosphate dehydrogenase
C0P5P9	33.3	9	0.70004375	Glycylpeptide N-tetradecanoyltransferase
A0A1D6N1P8	9.3	1	1.3151375	Phosphotransferase/hexokinase
A0A1D6DVJ7	30.4	13	0.744875	Plasma membrane ATPase
C0PGB5	11.1	4	0.717475	Pyruvate kinase
C0P6F8	44.1	47	0.54820625	Sucrose synthase
A0A1D6K2D8	23.3	14	1.32559375	Sucrose synthase2
C0PPB8	5.8	1	1.51045	UDP-glycosyltransferase 76C1
**Photosynthesis and Photorespiration**				
K7W104	6.6	1	1.4398125	2-methoxy-6-polyprenyl-1,4-benzoquinol methylase, mitochondrial
B6T144	39.1	3	1.30539375	B12D protein
Q6R9D5	3.4	1	1.34895	Cytochrome b
B6SPA1	49.3	3	1.36054375	Cytochrome b-c1 complex subunit 6
B6TEX6	69.8	11	1.324725	Cytochrome b-c1 complex subunit 7
B6TVC7	19.4	2	1.37949375	Ferredoxin
B4FYW4	22.7	2	1.45836875	Ferredoxin-3
B4FI05	16.8	3	1.33465625	Ferredoxin--NADP reductase
B4FRC8	56.5	49	1.38471875	Fruit protein PKIWI502
B4FFU4	16.4	1	1.338575	NADH dehydrogenase [ubiquinone] 1 beta subcomplex subunit 3-A
P04966	12.9	9	0.76561875	Photosystem I P700 chlorophyll a apoprotein A1
A0A1D6HY75	64.7	283	1.3460125	Photosystem I reaction center subunit IV A
B6TH55	62.6	277	1.36818125	Photosystem I reaction center subunit IV A
B6U534	23.9	9	1.40439375	Photosystem I reaction center subunit V
B4G1K9	23.5	9	1.3451375	Photosystem I reaction center subunit V
B6STG2	42.9	20	1.30215625	Photosystem I reaction center subunit XI
P24993	42.5	20	1.39320625	Photosystem II reaction center protein H
A0A1D6JR11	33.9	3	1.33035	Protein CutA chloroplastic
A0A1D6DT56	55	16	0.769575	Protochlorophyllide reductase1
B4FTR7	10.5	2	0.448975	Tab2 protein

^1^ accession No.: unique protein identifying number in the UniProt database; ^2^ coverage (%): the proportion of the number of non-repetitive amino acid numbers of all the peptides identified accounted of the total number of protein amino acids; ^3^ peptides (95%): the number of unique peptides identified by the confidence level greater than 95%; ^4^ fold change: is expressed as the ratio of intensities of up-regulated or down-regulated proteins between drought stress treatments and control (well-watered conditions).

## Supplementary Materials

The following are available online at https://www.mdpi.com/1422-0067/20/23/5956/s1.
