# Supplementary material for: iTRAQ-Based Proteomic Analysis Reveals Several Strategies to Cope with Drought Stress in Maize Seedlings
Source: Int J Mol Sci. 2019 Nov 26;20(23):5956. doi: 10.3390/ijms20235956 (PMC6928945; doi:10.3390/ijms20235956)
Supplement: Supplementary file 1 [file ijms-20-05956-s001.zip › Table S1 and S2 Changes in leaf length after drought treatment.docx]

| Table S1 Changes in soil moisture and leaf relative water content after drought treatment | | | | | |
| --- | --- | --- | --- | --- | --- |
|  | Soil moisture content | |  | Leaf relative water content | |
|  | Well watering (Control) | Stop watering (Drought) |  | Well watering (Control) | Stop watering (Drought) |
| Day0 | 52.67±1.71% ^A^ | 52.43±1.73% ^A^ |  | 92.50±0.70% ^a^ | 93.50±1.55% ^a^ |
| Day1 | 52.07±1.38% ^A^ | 38.65±3.67% ^B^ |  | 93.70±1.25% ^a^ | 92.20±1.42% ^a^ |
| Day3 | 52.14±1.39% ^A^ | 22.39±3.17% ^C^ |  | 93.77±2.18% ^a^ | 86.50±2.14% ^b^ |
| Day5 | 52.77±0.80% ^A^ | 15.05±2.11% ^D^ |  | 94.53±1.50% ^a^ | 68.70±4.26% ^c^ |
| Notes: Four replicates were performed. The values are presented as means ± SD. The letters denote significant differences (*p*< 0.05) based on Duncan’s multiple range tests. | | | | | |

| Table S2 Changes in leaf length after drought treatment | | | | | |
| --- | --- | --- | --- | --- | --- |
|  | Length of 3^rd^ leaf (cm) | |  | Length of 4^th^ leaf (cm) | |
|  | Well watering (Control) | Stop watering (Drought) |  | Well watering (Control) | Stop watering (Drought) |
| Day0 | 12.89±0.97^A^ | 12.77±1.00^A^ |  | 11.63±1.19^a^ | 11.82±1.19^a^ |
| Day1 | 15.86±0.80^B^ | 15.84±0.91^B^ |  | 14.63±1.05^b^ | 14.54±1.06^b^ |
| Day3 | 21.26±0.84^C^ | 21.03±0.88^C^ |  | 19.35±1.53^c^ | 19.37±1.54^c^ |
| Day5 | 25.88±1.05^D^ | 22.94±1.08^E^ |  | 24.43±1.59^d^ | 22.37±1.68^e^ |
| Notes: Four replicates were performed. The values are presented as means ± SD. The letters denote significant differences (*p*< 0.05) based on Duncan’s multiple range tests. | | | | | |
