# Supplementary figures and images for: iTRAQ-Based Proteomic Analysis Reveals Several Strategies to Cope with Drought Stress in Maize Seedlings
Source: Int J Mol Sci. 2019 Nov 26;20(23):5956. doi: 10.3390/ijms20235956 (PMC6928945; doi:10.3390/ijms20235956)

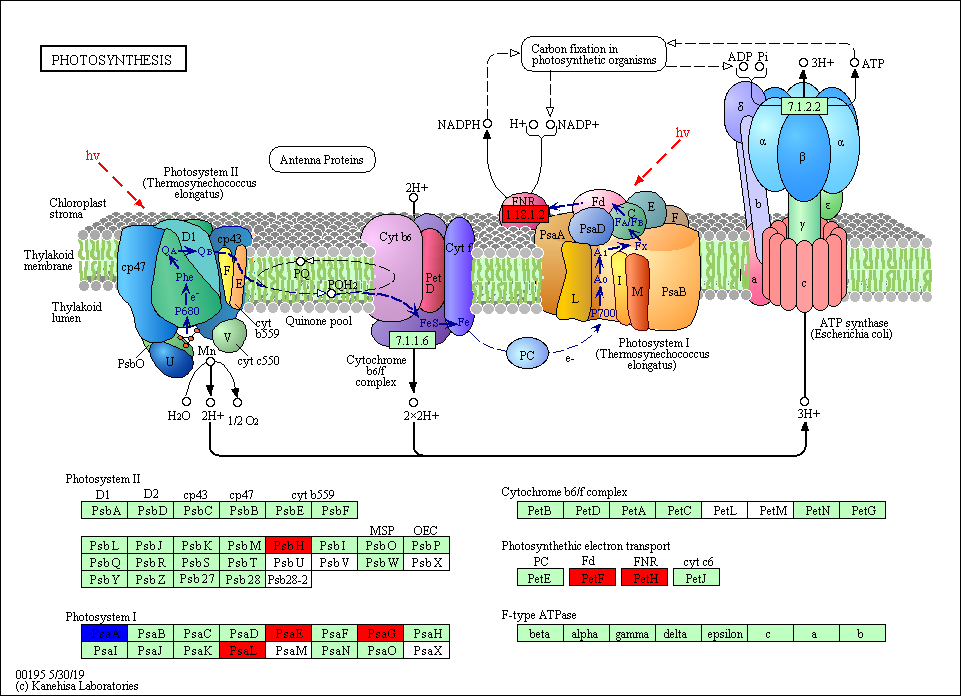

Supplement: Supplementary file 1 [file ijms-20-05956-s001.zip › Fig S1 KEGG.tif]
